# The exacerbated prevalence of acute malnutrition and growth retardation in Roma children living in camps

**DOI:** 10.1186/s13052-021-01122-4

**Published:** 2021-08-21

**Authors:** Rosaria Giampaolo, Rosaria Marotta, Francesco Saverio Biagiarelli, Antonella Zampa, Stefania Moramarco, Ersilia Buonomo

**Affiliations:** 1 Pediatric Emergency Department and General Pediatrics, Bambino Gesù Children’s Hospital, IRCCS, Rome, Italy; 2 Community of Saint’Egidio, Rome, Italy; 3 Department of Biomedicine and Prevention, University of Rome Tor Vergata, Rome, Italy

**Keywords:** Child malnutrition, Stunting, Wasting, Underweight, Immigrants, Roma, Camps, Italy

## Abstract

**Background:**

Child malnutrition is still a concern in marginalized groups of populations, such as immigrants living in very low socio-economic conditions. Roma children are within the most hard-to-reach populations, susceptible to undernutrition and growth retardation. In the city of Rome (Italy), the Hospital “Bambino Gesù”, in collaboration with the Catholic Association Community of Saint’Egidio, is dedicating free services for the health and nutritional needs of vulnerable people.

**Methods:**

A retrospective analysis was conducted on immigrant children visited at different ages (0–11 years old). Records including nutritional and growth assessment were collected from 2016 up to May 2020. Malnutrition was classified following the WHO 2006 standards. Data for Roma children living in extra-urban camps and non-Roma immigrant children living in urban areas were analyzed, odds ratios and univariate binary regressions were performed to investigate the risk of malnutrition within the two groups.

**Results:**

A sample of 414 children (57% under-five; 51.9% Roma), was included in the database. In the under-five children, underweight accounted for 7.6%, stunting for 11.7%, and wasting for 2.9%. The first year of life was the most crucial for nutritional status. Compared to the counterpart, Roma children accounted for nearly the total rate of malnutrition (wasting 4.8% vs. 1%; stunting 21.4% vs. 2%; underweight 14.2% vs. 1%). Univariate logistic regression confirmed under-five Roma children being at the highest risk of stunting at 12 months (OR: 16.1; CI 2–132; *p* = 0.01).

When considering the 176 school-aged children, undernutrition affected most Roma children (13% vs 1.9%), followed by stunting (5.8% vs 0.9%). Univariate logistic regression confirmed that Roma school-aged children were more likely to be underweight (OR: 7.8; CI 1.6–37.6; *p* = 0.01).

**Conclusions:**

Malnutrition in immigrant children is still of high concern in Italy. Its prevalence in Roma children living in extra-urban camps exceeds that of immigrant children living in urban areas and the rates of underweight, stunting and wasting of Roma children living in the Balkans. This exacerbating condition highlights the need of better assisting this fragile population that is at most risk of poverty, food insecurity and social exclusion in Italy, particularly during this pandemic crisis.

## Background

Malnutrition (undernutrition) refers to deficiencies in intakes, imbalance of essential nutrients or impaired nutrient utilization that may negatively affect growth in weight and height, as well as motor development [1, 2]. Compared to adults, children are particularly susceptible to undernutrition due to their high energy need per unit of body mass, limited energy reserves and the additional demands for growth [3].

According to the WHO, anthropometric malnutrition can occur in three broad forms: wasting, stunting and underweight [4]. Wasting is defined as low weight-for-height. It often indicates recent and severe weight loss, although it can also persist for a long time. It usually occurs when a person does not have adequate quality and quantity food or they have frequent or prolonged illnesses, such as diarrhea. Wasting in children is associated with a higher risk of death if not treated properly. Stunting is defined as low height-for-age and/or linear growth retardation. It is the result of chronic or durable undernutrition, usually associated with poverty, poor maternal health and nutrition, recurrent illness and/or inappropriate feeding and care in early life. Stunting impedes children from reaching their physical and cognitive potential, therefore impacting on human capital [5], reduces neurodevelopmental and cognitive function and increases the risk of chronic disease in adulthood, as reported by de Onis and Branca [6]. Childhood stunting is considered as the best overall indicator of children’s well-being; it is also an accurate reflection of social inequalities [6].

Recently WHO declared that in the world there are 47 million children under 5 years of age suffering from acute malnutrition, 14.3 million are severely wasted and 144 million are stunted [7]. The strong relationship between undernutrition and mortality is well known: around 30 to 54% of deaths among children under 5 years of age are linked to undernutrition [8]. Every country in the world is affected by one or more forms of malnutrition [9]. These mostly occur in low- and middle-income countries, while in high-income countries malnutrition is still occurring in marginalized groups of the population living in very low economic conditions [10, 11].

The Roma (often pejoratively called gypsies) are a heterogeneous ethnic group recognized as the most populous marginalized community in Europe in need of the greatest health services and access to food security [12]. Particularly impacted by poverty are those who arrived in Italy after the 1992–95 conflict in the Balkan area, since their houses were destroyed or occupied during the war. Most of them have been relegated into extra-urban camps since 1990 [13]. Data collected by the RSC Office of the Municipality of Rome Capitol during 2017–2019 reported that there are 9 authorized/tolerated camps where 4503 people live [14], while 6200 people are estimated to live in an unknown number of unauthorized camps [15].

Since 2016, the Hospital “Bambino Gesù” (OPBG) of Rome, in collaboration with the Catholic Association “Community of Saint’Egidio” (CSE), is dedicating a service to the health and nutritional needs of the most vulnerable immigrant children, specifically Roma children in authorized and not authorized urban and extra-urban camps in the city of Rome.

Specifically, every week a health mobile unit of the OPBG, offering pediatric care, reaches at least 3 Roma camps in extra-urban areas. In addition, also 2 peripherals Catholic Parishes located in urban areas host the mobile unit for visiting poor immigrant non-Roma children living in regular houses. Pediatric care included childhood vaccinations, clinical assistance for acute diseases, medication prescriptions, medical certificates for school access, nutritional assessment and growth monitoring, general counselling on child health promotion and eating habits when needed.

The CSE, in collaboration with OPBG, is offering free assistance for nutrition, health and education for Roma children living in these camps, where many children have no access to health care services, are not registered with the National Health Service and have no access to education at essential levels.

In this poverty context, the main objective of the project of the OPBG and CSE is to: assess and address malnutrition affecting Roma children during their first years of life; improve the quality and inclusiveness of health care services; supporting primary and secondary education.

The purpose of this study was to investigate the prevalence of acute and chronic malnutrition and linear growth retardation in a sample of Roma children living in authorized and unauthorized extra-urban Roma Camps, comparing data with those from a sample of non-Roma immigrant children living in regular houses.

## Methods

### Study population and setting

A retrospective analysis was conducted on pediatric medical records of immigrant children (0–11 years), who requested medical assistance and growth monitoring at the health mobile campers of the Hospital “Bambino Gesù” and at the CSE clinic, from 2016 to May 2020.

Children belonged to several hard-to-reach groups:

-first-generation immigrants (born in foreign countries then migrated to Italy with their families) and second-generation immigrants (born in Italy) living in regular houses in urban areas;

-Roma children born in families from the Balkan Countries and living in authorized and unauthorized camps in extra-urban areas.

### Anthropometric evaluation

At the time of the visits the anthropometric assessments included measurements of weight and length. Children were measured without clothing or footwear. For newborn babies and children weight (in kilograms) was determined using a mechanical baby scale (SECA 745—Class III). Length (in centimeters) was assessed by laying babies on their back and using a stadiometer for newborn babies and infants (SECA 210). Children above 2 years of age were measured standing to determine height (in centimeters) and were weighted using a portable scale with stadiometer (SECA 700).

Medical staff assessed the measurements, according to the WHO guidelines [16].

Anthropometric indicators were calculated using Anthro and Anthro Plus WHO softwares [17]. Nutritional status was defined using the standardized anthropometric z-scores as per WHO 2006 standards [18]: length-for-age z- score (LAZ), weight-for-length z-scores (WLZ), weight-for-age z-score (WAZ) for under-five aged children; BMI-for-age z-score (BAZ) for children above 5 years.

Following WHO criteria, general malnutrition was defined as: stunting/LAZ < -2, wasting/WLZ < -2, and underweight/WAZ < -2 in under-five aged children. For children above 5 years of age, BAZ < -2 identified underweight, while BAZ > 2 overweight.

Data for children was grouped according to the age at time of the visit. Information was analyzed at birth, 6 months, 12 months, 24 months, 36 months, 48 months, 59 months, then between 6 and 10 years of age.

### Statistical analysis

WHO Anthro and Anthro Plus Software (Version 3.2.2, January 2011) were used to compute children’s weight-for-age WAZ), weight-for-length (WLZ), length-for-age (LAZ), BMI-for-age (BAZ) z-scores.

A database was generated without names, but children were given an identification number to ensure their privacy. The database was analyzed using the SPSS software system 21.0 (IBM, Somers, NY).

Descriptive data and variables measured were presented as means with standard deviations (SD).

The Student’s t-test was performed to assess the statistical significance of differences between continuous variables for children living in Roma camps vs. immigrants not living in camps. The Chi-squared test was performed to compare proportions in prevalence of malnutrition between the two groups.

Univariate binary regressions were performed to investigate the risk for under-five Roma children of being stunted at 12 months, and the risk of being underweight when school-aged (OR; 95% CI).

Given the anonymous collection of data the Ethical Committee of the coordinating center advised that ethical approval was not necessary.

## Results

A sample of 414 children, for a total of 579 anthropometric data, was analyzed according to the age at the time of visit. The majority of them did not have access to the Public Health System. Mean mothers’ age was 24.7 years ±3.9 (24.2 years ±4.2 for Roma vs. 25.4 years ±3.8 for non-Roma; *p* = 0.035). The 92% of the mothers of Roma children were illiterate and not confident with Italian language. A similar background was found for non-Roma mothers, despite specific information were not collected.

The 46.7% of Roma families had more than 4 children living in the same household, compared to the 9.7% of non-Roma families (*p* < 0.001). All the Roma children were living in camps in extremely poor living conditions, particularly not having access to running water and sanitation. These conditions were not found in non-Roma immigrants, since they were living in regular houses.

The rate of breastfeeding in the sample of under-five children was of 71.4%. When considering the two groups, the rate of breastfeeding was 90.2% in non-Roma children (mean 14.23 months ±11.5 SD) vs. 51.2% in Roma (mean 13.4 months ±13.9 SD).

When considering the overall sample of under-five aged children (403 anthropometric data; 50.1% Roma; 50.4% males), underweight accounted for 7.6%, stunting for 11.7%, and wasting for 2.9%. As expected, Roma children living in extra-urban camps accounted for nearly the total rate of under-nutrition: respectively, the prevalence of acute malnutrition or wasting was 4.8% vs. 1% in the counterpart, whilst the prevalence of stunting was 21.4% vs. 2%. The prevalence of underweight was 14.2% vs. 1%. More specifically, Table 1 presents anthropometric measurements at birth, then divided by different ages. Weight and length at birth were referred by the mothers for 76.6% of the total sample. The first year of life appeared to be the most crucial from the point of view of nutrition and growth. With reference to the WAZ trend, the values remained below the median over the months with a negative peak at 6 months. Similar results at all ages were found for LAZ. The median of WHZ was close to normal value, probably due to the impact of the negative value of LAZ.

**Table 1:** Anthropometric measurements of under-five children grouped by months (all sample)

	At birth	3–6 months	7–12 months	13–24 months	25–36 months	37–48 months	49–59 months
**Weight, mean SD (Kg)**	3.01 ± 0.46	7.1 ± 1.0	8.9 ± 1.3	11.8 ± 1.5	13.8 ± 1.9	15.9 ± 2.4	18.0 ± 3.1
**Height, mean SD (cm)**	49.4 ± 2.2	64,6 ± 2.8	72.9 ± 3.5	84.5 ± 4.0	93.4 ± 4.4	100.3 ± 5.0	107.2 ± 5.1
**Weight for age z-score (WAZ)**	−0.64 ± 1.0	− 0.74 ± 1.0	− 0.49 ± 1.6	− 0.11 ± 1.1	−0.21 ± 1.1	−0.27 ± 1.2	−0.22 ± 1.1
**Length/Height for age z-score (LAZ/HAZ)**	0.47 ± 1.2	−1.00 ± 1.0	− 0.92 ± 2.0	− 0.70 ± 1.2	−0.56 ± 1.1	−0.69 ± 1.2	−0.52 ± 0.9
**Weight for height z-score (WHZ)**	−0.85 ± 1.2	0.04 ± 1.0	− 0.06 ± 1.0	0.32 ± 1.0	0.15 ± 1.0	0.2 ± 1.0	0.09 ± 1.1

Table 2 investigates the differences in anthropometric indices between under-five Roma and non-Roma immigrant children, at the same age. Compared with their counterpart, Roma children presented the lowest values of anthropometric measurements, starting with the referred measurements at birth, then confirmed in all ages. Significant differences were found for WAZ and LAZ/HAZ between the two groups. Even WHZ was lower in Roma children at all ages, despite not being statistically significant.

**Table 2:** Anthropometric measurements of under-five children grouped by months (Roma vs. non-Roma)

Anthropometric measurement,1 mean ± SD	Roma children	Non-Roma children	Student t-test (***p***-value)
**At birth**			
**Weight for age z-score**	−0.87 ± 1.0	− 0.29 ± 0.9	0.000
**Length for age z-score**	− 0.34 ± 1.1	0.33 ± 1.1	0.001
**Weight for height z-score**	−0.96 ± 1.3	− 0.71 ± 1.2	NS
**3–6 months**			
**Weight for age z-score**	−1.02 ± 1.0	− 0.32 ± 0.9	0.015
**Length for age z-score**	−1.42 ± 0.7	− 0.38 ± 1.1	0.001
**Weight for height z-score**	−0.05 ± 1.1	− 0.02 ± 1.1	NS
**7–12 months**			
**Weight for age z-score**	−0.99 ± 1.2	− 0.00 ± 0.8	0.000
**Length for age z-score**	−1.65 ± 0.9	− 0.23 ± 0.9	0.000
**Weight for height z-score**	− 0.15 ± 1.3	0.08 ± 1	NS
**13–24 months**			
**Weight for age z-score**	−0.56 ± 1.2	0.25 ± 0.9	0.002
**Length for age z-score**	−1.4 ± 1.0	−0.1 ± 1	0.000
**Weight for height z-score**	0.23 ± 1.1	0.4 ± 1	NS
**25–36 months**			
**Weight for age z-score**	−0.74 ± 1.1	0.17 ± 0.9	0.001
**Height for age z-score**	−1.34 ± 0.9	0.01 ± 0.9	0.000
**Weight for height z-score**	0.1 ± 1	0.25 ± 1.1	NS
**37–48 months**			
**Weight for age z-score**	−0.66 ± 1.1	0.10 ± 1.2	0.006
**Height for age z-score**	−1.29 ± 0.9	− 0.10 ± 1.2	0.000
**Weight for height z-score**	0.26 ± 1	0.31 ± 1	NS
**49–59 months**			
**Weight for age z-score**	−0.32 ± 1.1	− 0.07 ± 1.1	NS
**Height for age z-score**	−0.96 ± 0.8	0.09 ± 0.8	0.000
**Weight for height z-score**	0.13 ± 1.3	−0.5 ± 0.7	NS

As regards low birthweight (<−2.5 kg), odds ratio between Roma and non-Roma children was calculated and the risk of being low birth weight increased by 2.8 times in Roma children as shown in Table 3.

**Table 3:** Risk of having low birthweight (Roma vs. non-Roma children)

	Roma n. (%)	Non-Roma n. (%)	OR (CI)
Birth weight < 2.5 kg	28 (19.2)	7 (7.6)	2.8 (2.2–6.9)
Birth weight > 2.5 kg	118 (80.8)	85 (92.4)	

Univariate logistic regression showed that Roma children under the age of five had the highest risk of being stunted at 12 months of age (OR: 16.1; CI 2–132; *p* = 0.01).

As regards the school-aged children, from 6 to 11 years, the sample included 176 children (39.2% Roma; 54% males). Table 4 presents their anthropometric data, grouped for ages at time of visit. The WAZ values were negative in the oldest children, while the HAZ values were constantly negative. BAZ values were positive and close to the median values of reference.

**Table 4:** Anthropometric characteristics of school-aged children at different time of visits

	6 years	7 years	8 years	9 years	10 years	11 years
**Weight, mean** ± **SD (Kg)**	19.9 ± 6.4	23.5 ± 5.8	24.7 ± 6.8	28.3 ± 9.7	29 ± 4.9	38.5 ± 10.6
**Height, mean** ± **SD (cm)**	110.9 ± 5.9	119.6 ± 7.1	120.9 ± 6.6	126.9 ± 7.5	131.9 ± 5.6	143.7 ± 7.7
**Weight for age z-score (WAZ), mean** ± **SD**	0.1 ± 1.9	0.41 ± 1.3	0.18 ± 1.6	0.16 ± 1.9	− 0.11 ± 0.8	−1.48 ± 1.5
**Height for z-score (HAZ), mean** ± **SD**	− 0.07 ± 1.1	0.22 ± 1.2	− 0.28 ± 1.1	− 0.26 ± 1.2	−0.35 ± 0.8	0.16 ± 1
**BMI for age z-score (BAZ), mean** ± **SD**	0.16 ± 2.2	0.38 ± 1.1	0.45 ± 1.6	0.41 ± 1.9	0.10 ± 0.8	0.32 ± 1.4

Table 5 reports the differences in anthropometric measurements between Roma and non-Roma school-aged children. There were significant differences in values of body weight (*p* = 0.002), height (*p* = 0.004), WAZ (*p* = 0.001), HAZ (*p* < 0.001) and BAZ (*p* = 0.004) within the two groups.

**Table 5:** Anthropometric characteristics of school-aged children (all sample, Roma and non-Roma)

	Total (n.177)	Roma	Non-Roma	***p***-value
**Age, mean** ± **SD (months)**	93.3 ± 17.3	94 ± 13	92 ± 19	NS
**Weight, mean** ± **SD (kg)**	26.8 ± 8.7	24.3 ± 7.3	28.3 ± 9.1	0.002
**Height, mean** ± **SD (cm)**	123.7 ± 10.5	122.0 ± 9.4	126.5 ± 10.7	0.004
**Weight for age z score (WAZ), mean** ± **SD**	0.15 ± 1.5	− 0.38 ± 1.6	0.50 ± 1.4	0.001
**Height for age z-score (HAZ), mean** ± **SD**	−1.15 ± 1.1	−0.61 ± 1.1	0.14 ± 1.1	< 0.001
**BMI for age z-score (BAZ), mean** ± **SD**	0.3 ± 1.6	0.04 ± 1.5	0.5 ± 1.5	0.04
**Underweight (WAZ < -2) (%)**	6.3	13	1.9	0.004
**Underweight (BAZ < -2) (%)**	2.3	4.3	0.9	NS
**Stunting (HAZ < -2) (%)**	2.8	5.8	0.9	NS
**Overweight (BAZ > 2) (%)**	10.8	7.2	13.1	NS

When considering the whole sample, undernutrition was confirmed as the form of malnutrition affecting most Roma children, with underweight accounting for 13% (vs 1.9% of non-Roma) and stunting accounting for 5.8% (vs 0.9% of non-Roma), despite the latter not being statistically significant. Conversely, the prevalence of overweight in non-Roma children nearly doubled that of Roma children (13.1% vs. 7.2%; but not statistically significant). Univariate logistic regression showed that Roma school-aged children were more likely to be underweight (OR: 7.8; CI 1.6–37.6; *p* = 0.01).

## Discussion

**Fig. 1:**
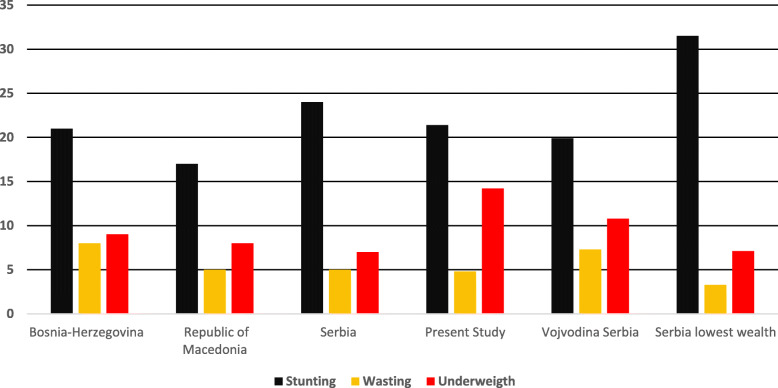
Prevalence of malnutrition in under-five Roma children in different studies

The data reported in this study represents a sample of vulnerable immigrant children living in extremely poor settings in Rome, the capital city of Italy. The vulnerability of this population, referring to nutritional status, is already well known in literature, with immigrants likely to be most exposed to malnutrition, especially under-nutrition [19, 20]. Our data highlights the high prevalence of under-nutrition in Roma children living in authorized and unauthorized extra-urban settings since their birth. At the time of birth, our sample showed already a 19.2% prevalence of low weight (<− 2.5 kg), confirming that Roma infants are more likely to be under-nourished weight than non-Roma [21]. The UNICEF’s report showed that in Bosnia and Herzegovina the proportion of low birth weight for Roma infants was over four times that of non-Roma, being around 14% in 2015. As well known, newborn’s weight is a good indicator of a mother’s health and nutritional status, as well as of their chances for survival, growth, long-term health and psychosocial development. Consequentially the high prevalence of low birthweight reported in the present study might be a significant risk factor associated with poor child development outcomes. As regard malnutrition in Roma under-five children, in Fig. 1 we have compared our findings with the results from some other studies, reported by the UNICEF, conducted in different Balkan settlements [21]. The present study reports similar data on chronic malnutrition and highest prevalence of acute malnutrition, in particular underweight. Our results confirm that Roma children under the age of five are most vulnerable to under-nutrition.

The highest rate of malnutrition, especially in Roma children, was observed for chronic malnutrition-stunting, in agreement with other published studies in Eastern Europe [21]. Levels of chronic malnutrition in our group is comparable to the Roma Serbian’s study [22, 23], as well as some low- and middle-income countries [24]. The faltering in linear growth of Roma children under-five is an expression of protracted food insecurity, and a lack of the mothers’ knowledge in feeding practices in the first years of age. In the specific context of extra-urban camps, the lack of access to basic services, the economic barriers, the poor mothers’ education are the main drivers of child malnutrition [25].

A very high prevalence of underweight was found among Roma children (14%), nearly doubling that reported in the Serbian study, with a similar prevalence to that of the Vojvodina region (10.8%) [22]. Our findings are comparable with the level of underweight in low-middle income countries, where the World Bank reports a prevalence of 14.5% in 2019 [26].

As expected, even the prevalence of wasting was significantly higher in Roma children, similar to the Serbian study, emphasizing the disparity in terms of poverty and health conditions between Roma children living in camps and non-Roma living in urban settings.

The trends in malnutrition for under-five children reflect findings of a previous study conducted in a similar population in the city of Rome, which highlighted that the Roma children presented all anthropometric indices significantly lower with respect to the entire immigrant population [27].

A recent study, conducted in Italy on immigrant children reported that children living without access to basic water and sanitation services, as well as household commodities (i.e. heater, refrigerator, electricity) increased the risk of these children being stunted [28]. The prevalence of malnutrition was confirmed even in the school aged children, with significant differences when considering the two kinds of malnutrition: under-nutrition and over-nutrition. Compared to the youngest group, for Roma children the risk of being underweight was confirmed, while a lower rate of stunting was detected, with a prevalence at school-age of 5.8%. School-aged immigrant children presented the lowest rate of underweight, comparable to rates found for Italian children living in socioeconomic inequality contexts, as reported by Buonomo et.al [28].

**Fig. 2:**
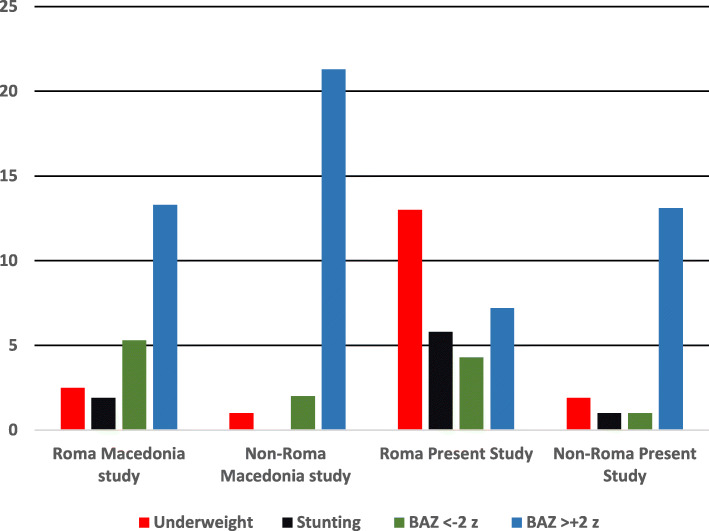
Prevalence of malnutrition in school-aged children: comparison with the Macedonia study

Our findings exceed the results of a similar study conducted in the Republic of Macedonia (Fig. 2), that reports the lowest level of malnutrition among Roma school-aged children, versus non-Roma [29].

Underweight was detected in the Republic of Macedonia in 1.9% of Roma children vs. 0.7% of non-Roma, while stunting was assessed in only 1.9% (vs. 0%). When considering BAZ < -2, our findings were quite similar to the Macedonia study, where the 4.8% of Roma children were affected by the low BMI for age (vs. 1.8% non-Roma). The same study found a prevalence of chronic malnutrition in Roma children of 2%.

As regards overweight, the Macedonia study reports a prevalence of 19.5% in first grade school-aged non-Roma children (6–9 years) vs the 8.1% in Roma children. As regards the latter, similar results were found in our study.

All our findings underline that in Roma camps the main challenges in protracting and exacerbating people segregation is the persistent food insecurity related to poverty, lack of food and mothers’ level of education which, as recently reported by Stamenkovic et al., is one of the main factor impacting of children nutritional status [30].

The COVID-19 pandemic is exacerbating food insecurity in marginalized populations and researchers are calling policy makers for more nutrition-related assistance [31]. Further investigations, including maternal education and household poverty as predictors of malnutrition, coupled by early assessment of children growth velocity [32], are essential to better understand the role of food insecurity and food habits in this underprivileged population.

### Limitations of the study

Data of the study was collected during pediatric field visits of children seeking clinical assistance for health diseases, acute conditions, medical prescriptions, nutritional and growth monitoring, childhood vaccinations, and medical certificates for school access. However, the current study mainly focused on nutritional assessment and growth monitoring. Socio-demographic characteristics were not available for the entire sample. No information on food habits, both for children and families, as well as maternal health was collected for the purpose of this study. Further studies will be conducted to fill in these gaps.

## Conclusions

There is a higher prevalence of both acute and chronic under-nutrition among Roma children living in authorized and unauthorized extra-urban camps in the Municipality of Rome, compared to immigrants living in regular houses in urban areas.

The high rates of underweight, stunting and wasting, exceeded the malnutrition indicators for countries of provenience, highlighting the need to better assist fragile populations that are at highest risk of poverty, food insecurity and social exclusion. Given its adverse effects on short and long-term clinical outcomes, especially taking into account the ongoing Covid-19 pandemic, malnutrition should be prevented, and must be promptly recognized and treated properly.

In conclusion, the authors underline the need of political commitment and knowledge at national and international levels to reduce social inequities, overcoming the segregation in Roma camps of these underserved populations, therefore facilitating their inclusion in the society [23, 33].

## Data Availability

Not applicable.

## Abbreviations

BAZ: BMI for age z-score
HAZ: Height for age z-score
LAZ: Length for age z-score
OR: Odds ratio
WAZ: Weight for age z-score
WHZ: Weight for height z-score
